# Progress and Challenges in China Turfgrass Abiotic Stress Resistance Research

**DOI:** 10.3389/fpls.2022.922175

**Published:** 2022-06-14

**Authors:** Lai Jiuxin, Han Liebao

**Affiliations:** School of Grassland Science, Beijing Forestry University, Beijing, China

**Keywords:** turfgrass, temperature tolerance, drought tolerance, molecular breeding, industrial development

## Abstract

Turfgrasses are an important vehicle for urban ecology and one of the most important indicators of economy and civilization. The biological characteristics of different turfgrass species affect the productivity and quality of the turf and its potential use in landscapes, slopes, and sports fields. Cultivation and management techniques can assist turfgrasses to meet the challenges of climate change, while the development of molecular breeding will provide a broader platform for the application of turfgrasses. The turfgrass industry of China has developed considerably in the last three decades; however, there is still an objective gap with developed countries. This manuscript reviewed the research progress of turfgrass resistance breeding, analyzed the bottlenecks in the development of turfgrass resistance breeding, and put forward the strategies to cope with the bottlenecks. Our review aims to promote research and utilization of turfgrasses.

## Introduction

Turfs are relatively flat, evenly vegetated grasslands dominated by low perennial herbaceous plants that have been artificially planted or naturally formed and are artificially managed for landscaping, environmental protection, and sports ground. Turfs usually have a specific purpose and strongly intervene while also assuming a unique ecosystem function. China’s urban development, especially the goal of developing into a strong sports nation, has led to an increased interest and demand in high quality and resilient sports turfs. However, this momentum provides new challenges and opportunities for the development of the turf industry. This paper reviews the research progress on turfgrasses and proposes a way to develop the turfgrass industry.

Turfgrass is used to grow turf that together with the soil, microorganisms, and the environment, form an organic ecosystem of the lawn. Turfgrass forms a dense even turf if mown and maintained properly. There are many different types of turfgrasses and these are classified according to a classification criterion based on their characteristics. Gramineous plants used to play a major role in the establishment of lawns, but in the recent years non-Gramineous species, such as *Salicaceae*, *Leguminosae*, and *Spiniferae* have also been introduced. They are also classified as thin-leaved and broad-leaved on the basis of the width of the grass leaves or as low and prostrate or tall and robust on the basis of turfgrass height. Moreover, based on their use turfgrasses are classified into ornamental, regular green, soil retaining, and accent turfgrass. However, they are mostly classified as, warm-season and cool-season turfgrasses, based on the climatic conditions of their geographic location. Cool-season turfgrasses, also known as winter grasses, mainly include *Poa L*., *Agrostis L.*, *Festuca L*., and *Lolium L*. and are mostly found in temperate to subtropical regions of the Northern Hemisphere and are suitable for planting in northeast, north, northwest, and southwest China (Han, 1996). Whereas, warm-season turfgrasses, also known as summer grasses, are mainly found in tropical and subtropical regions with warm humid, warm semi-humid, and warm semi-arid climates, as well as in the central temperate regions of China. Warm-season turfgrasses are primarily limited by extremely low temperatures and duration. This type of grass shows high heat resistance with an optimum growth temperature of 25°C–35°C, disease resistant and tolerates rough management. The main species in this group are *Cynodon L.*, *Zoysia L.*, *Axonopus L.*, *Eremochloa L*., *Buchloe Engelm.*, etc.

China is rich in turfgrass germplasm resources. However, few new turfgrass varieties are being cultivated, most of which have been imported from the U.S. The cultivation of turfgrass in China began in 1950, with the introduction of buffalo grass in Beijing. Additionally, small quantities of seeds of *Poa Annua*, *Agrostis canina*, and *Lolium perenne* were imported in the late 1980s (Gao and Wang, 2000) and in 1984, “Kentucky” and “Wabash,” varieties of *P. Annua*, were introduced in Qingdao; these varieties had a long green period, high resistance, excellent lawn quality, and were low maintenance (Shao and Liu, 1995). In 1987, many species of *Agrostis* were first introduced in Henan and after years of cultivation, they have obtained a cool-season turfgrass, *Agrostis stolonifera*, suitable for areas north of the Yellow River. Further, more than 10 grass species suitable for desert planting were obtained in trials conducted by Lv (1995) and Liu et al. (1998) in arid and deserted areas.

**Figure 1:**
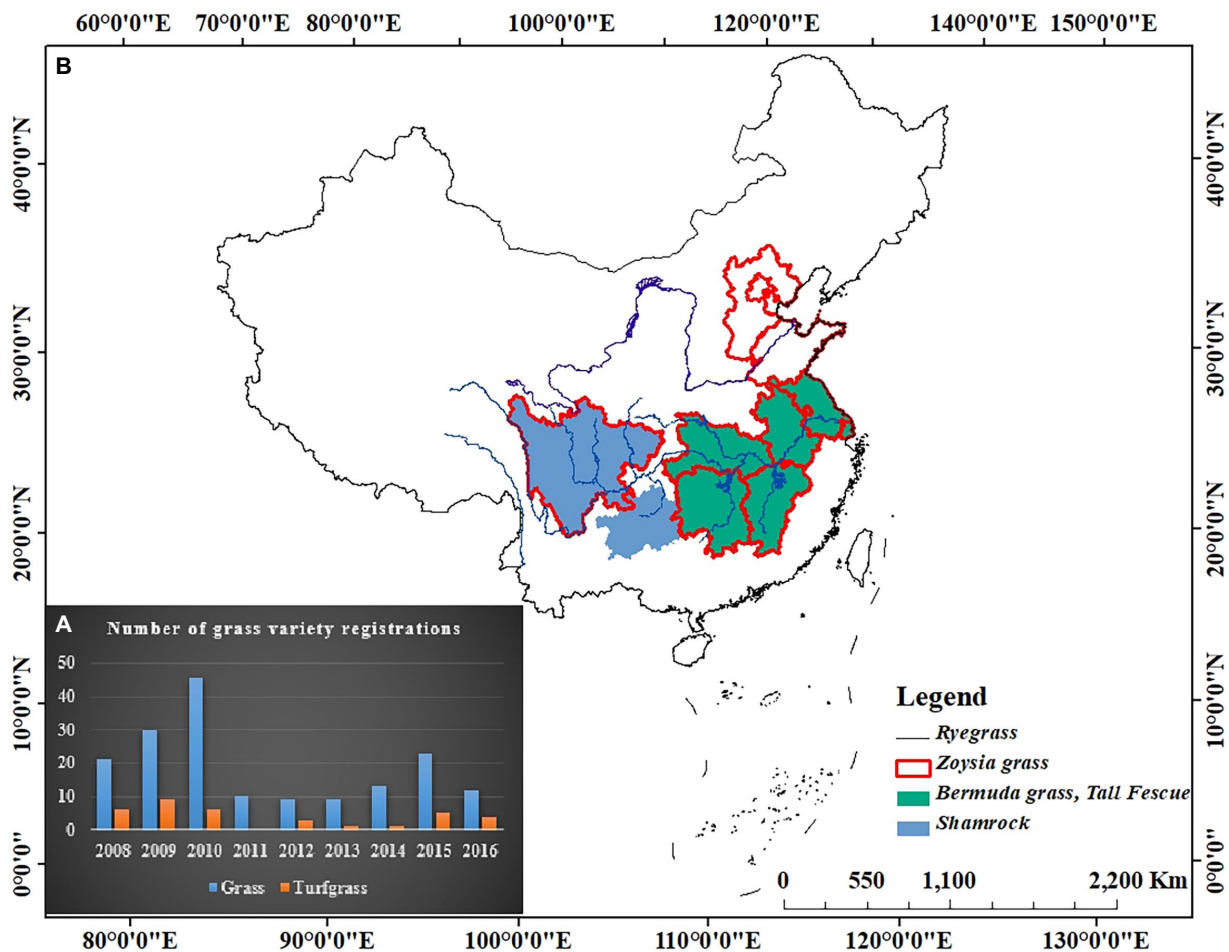
Variety registrations of turfgrass from 2008 to 2016 in China. **(A)** Number of turfgrass variety registrations; **(B)** Distribution of registered varieties.

Research on native turfgrass germplasm resources started late in China, but the collection and evaluation of germplasm resources have advanced in the recent years. Dong and Gong (2001) collected more than 100 turfgrass resources from different areas in China and Weng (1999) collected 147 accessions of germplasm of the genus *Zoysia* in Taiwan. The Institute of Turfgrass Research, Beijing Forestry University collected 132 accessions of *Zoysia*, which are naturally distributed in China (Wang et al., 2006). The Jiangsu Institute of Botany, Chinese Academy of Sciences, collected 1,227 accessions of eight genera and 20 species of warm-season turfgrasses in China (Liu, 2011). Although the collection of turfgrass germplasms has been on par with foreign countries, breeding of new turfgrass varieties in China is still relatively lagged behind. The registration of grass varieties in China is increasing every year, with 173 varieties registered between 2008 and 2016 (Figure 1), of which one-third are being utilized. Most of the registered varieties are types of forage grass or ecological grass. However, turfgrass varieties are still relatively few and the rich germplasm resources of turfgrasses in China are not fully utilized, the distributions of registered varieties were illustrated in Figure 1. The development of molecular breeding technology has shortened the plant breeding cycles at an accelerated rate. However, most of the fundamental research materials used in turfgrass molecular biology research are imported varieties. Therefore, our primary task is to strengthen the in-depth research and application of turfgrass resources, actively use molecular methods and clone key genes for resistance breeding, also for accelerating the pace of selection and breeding of new varieties of turfgrass.

## Current State of Research on the Stress Tolerance of Turfgrasses

**Figure 2:**
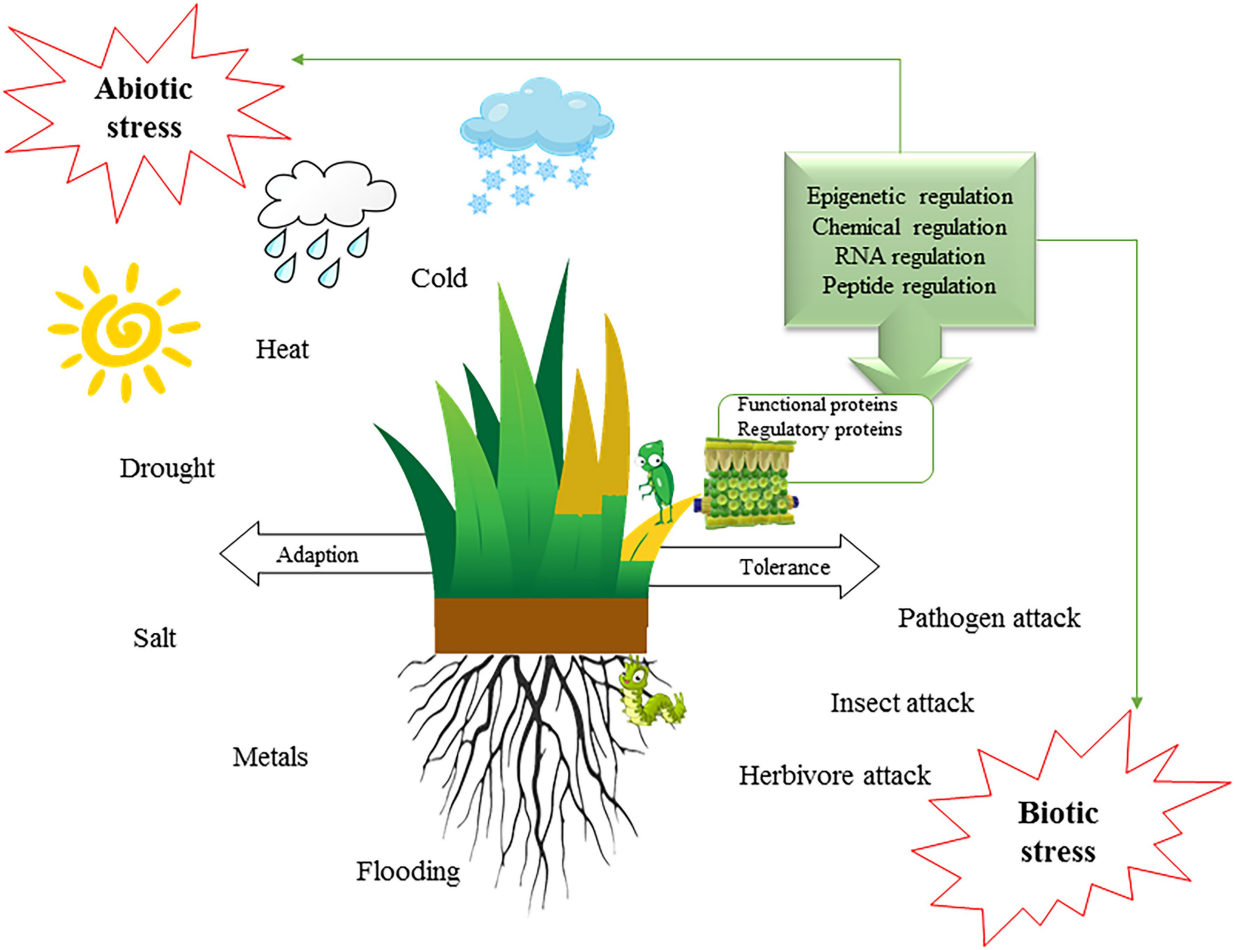
Tolerance and response to environmental stress in turfgrass.

Turfgrass is popular for its carpet-like appearance. It is used on sports fields and is an important component of landscaping; it further helps resist flooding and storm erosion. In hot areas, well-maintained turfs can naturally reduce the temperature thereby reducing urban cooling energy consumption. However, there are many challenges to maintain a healthy turf. Most turfgrasses are susceptible to extreme weather, disease, pests, and soil salinity, which can lead to brown spots or turf death (Figure 2).

### Research on Heat and Cold Tolerance of Turfgrasses

The regulation of heat and cold tolerance in turfgrass is mainly based on their maintenance (fertilization, watering, etc.) or the use of advanced technologies (transgenic for heat and cold tolerance), which can effectively reduce the damage caused by temperature adversity. The underlying principle of turfgrass research is to reduce the accumulation of oxygen radicals and toxic substances in plants, reduce the degree of oxidative stress, prevent lipid membrane peroxidation, reduce protein denaturation, and improve photosynthetic efficiency in turfgrasses. The fundamental purpose of turfgrass research is to promote the orderly functioning of plant physiological metabolism even under extreme conditions.

Under low temperature, many physiological changes occur in turfgrasses, for example, increase in soluble protein concentration, starch and non-structural carbohydrates, sulfur and hydroxyl nitrogen and amino acid contents, as well as photosynthetic efficiency and amylase activity and decrease in hydration levels (Xu et al., 2011). Warm-season turfgrasses are less resistant to cold; low temperatures reduce photosynthesis and respiration in these varieties. The limitations caused by extreme temperature can be compensated for by management techniques such as reseeding. Yan et al. (1998) chased ryegrass on hybrid bermudagrass in autumn to compensate for the withering weakness of hybrid bermudagrass in winter, eventually achieving evergreen landscape turfs in all seasons in Shanghai People’s Square. Use of a mixed variety of ryegrass was suggested over direct planting of cool-season grasses to reduce initial construction costs and year-round management costs.

Proper application of nitrogen and potassium fertilizers can help enhance heat or cold tolerance of turfgrass and prolong the green period. Application of phytohormones, such as gibberellin (Hedden and Sponsel, 2015), abscisic acid (Li et al., 2017), exogenous spermine (Pegg, 2014), and ethylene (Hu et al., 2020) can also effectively enhance stress tolerance of turfgrass. Some endophytic fungi increase drought and heat tolerance of turfgrasses by altering antioxidant enzyme activities (Xu et al., 2017a). Temperature tolerance of different grass species varies greatly; the variety “Midiron” has a semi-lethal temperature of −11°C, hybrid bermudagrass ‘Brooking’ is able to tolerate temperatures as low as −17°C, while the wild bermudagrass in Xinjiang can survive at −32°C. Traditional breeding methods can produce grasses with improved resistance; however, they have long breeding times and disorderly sample selection. The breeding cycle can be accelerated and the target characteristics can be clearly defined using cell and molecular engineering. Somatic cell-induced mutagenesis, or the introduction of functional genes into the tissues or protoplasts of specific plants, can create highly resistant transgenic plants and has been widely reported in crops such as rice and maize. However, successful use of transgenic variety in turfgrass is yet to be achieved.

The scientists from Wuhan Botanical Garden analyzed 106 germplasm resources of bermudagrass for cold tolerance and found 34 loci related to cold tolerance traits using SSR molecular markers. They identified that the *CdERF1* gene was suppressed by low temperature using the virus-induced gene silencing (VIGS) method. RNA-seq analysis in overexpressed transgenic *Arabidopsis* and the roots of VIGS bermudagrass revealed that *CdERF1* might respond to low-temperature stress by transcriptionally regulating the expression of antioxidant genes (POD), lipid transfer protein family genes (LTP) and CBF2 genes (Hu et al., 2020). Exogenous ABA-mimicking ligand treatment can significantly increase cold tolerance by modulation of stress-inducible genes in *Cynodon dactylon* (Cheng et al., 2016).

Alam et al. (2018) characterized *FaHSFA3*, *FaAWPM*, and *FaCYTC2* genes in *Festuca arundinacea* in response to heat stress and improved its thermotolerance using melatonin and 24-epibrassinolide. In addition, Sun et al. (2016) found that small heat shock proteins are involved in abiotic stress response and cloned *AsHSP17* gene from creeping bentgrass to study its role in stress response. *AsHSP17* encodes a 17 kDa protein whose expression is strongly induced by heat in leaves and by salt and abscisic acid (ABA) in roots. *AsHSP17* transgenic *Arabidopsis* plants were strongly sensitive to heat and salt, along with reduced chlorophyll content and reduced photosynthesis compared to the wildtype plants. Overexpression of *AsHSP17* in creeping bentgrass led to high sensitivity to exogenous ABA and salt during germination and growth. Further, *AsHSP17* was shown to act as a protein chaperone that negatively regulates photosynthesis and ABA-dependent independent signaling pathways in response to adverse environmental stress (Sun et al., 2016). To further validate the response of heat shock proteins to stress in *Arabidopsis*, Han’s group cloned a novel chloroplast heat shock protein gene, *AsHSP26.8a* whose expression is strongly induced by heat in both leaves and roots. Transgenic *Arabidopsis* plants overexpressing *AsHSP26.8a* showed reduced tolerance to high temperatures. Whole gene expression analysis revealed that *AsHSP26.8a* regulates the expression of heat stress transcription factors. This result suggests that *AsHSP26.8a* may negatively regulate plant responses to biological stresses by regulating ABA and other signaling pathways (Sun et al., 2020).

### Turfgrass Drought Tolerance Study

**Figure 3:**
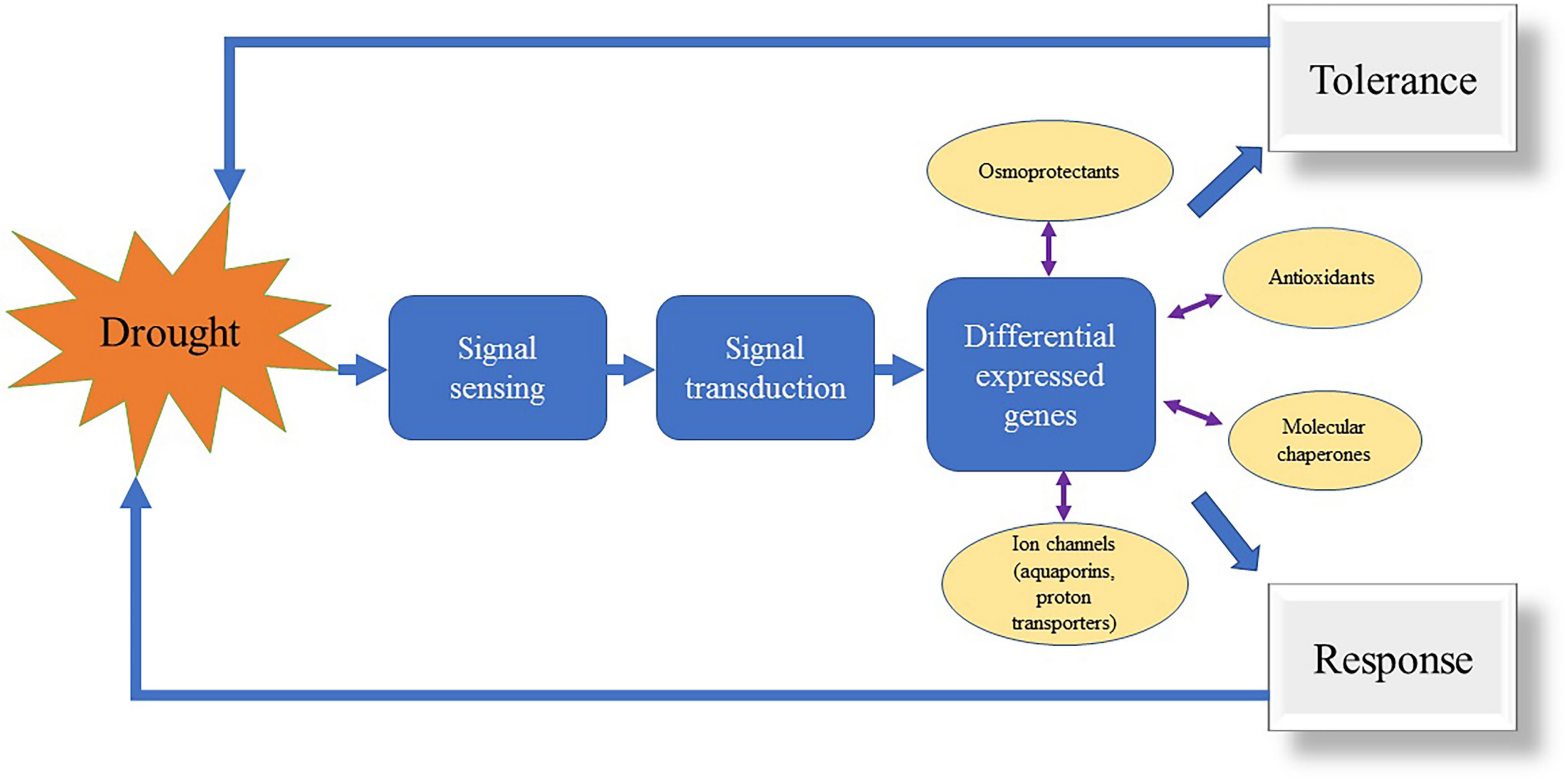
Drought response mechanism in turfgrass.

In the last decade, DNA sequencing technology has developed rapidly and the cost of sequencing is decreasing day after day, with increasing applications on whole genomes, chromosomes, regional genomes, and specific genes. The molecular regulatory mechanisms of plants in response to stress are complex such as for drought stress (Figure 3). Many genes have been reported in plants in response to drought stress (Wang et al., 2016; Ma, 2017). However, the identification of each gene from the gene bank is very laborious. Ma (2017) used GWAS combined with RNA-seq approach for differential gene expression analysis in a natural population and screened a total of 46 candidate genes associated with drought stress in plant leaves. Apart from molecular marker development, next-generation sequencing (NGS) technologies can be applied for resequencing and identifying domestication-related genes by comparing crop genomes with their wild relatives (Henry, 2012). Further, NGS can be used in plant breeding by using genome-wide selection studies to predict the reproductive value of traits having high potential to develop economical varieties. Thus, NGS has become a powerful tool for marker-based detection of DNA sequence polymorphisms and is a powerful tool for the next generation of plant breeding. Furthermore, many studies have reported on the response of herbaceous plants to stress such as drought using RNA-seq, WGRS, and GWAS to identify drought-related molecular markers in plants. However, only a few resistance-related molecular markers were discovered in turfgrass. Ontology-based studies on resistance mechanisms have rarely been done on turfgrass. Additionally, most research on resistance mechanisms has identified the target genes in model plants and transformed functional genes in turfgrasses whose metabolic pathways have been revealed in model plants (Evers et al., 2010; Tůmová et al., 2018). The water consumption of turfgrasses has been a major concern for applications in urban landscapes and sports fields. Approximately 26% of changes in environmental factors trigger plants into drought-responsive growth patterns (Obidiegwu et al., 2015). Xu et al. (2011) used drought and rehydration treatments to compare the antioxidant enzyme levels and transcript levels in *P. pratensis* “Midnight” and “Brilliant” varieties. The results revealed that the activities of APX, monodehydroascorbate reductase, glutathione reductase, and dehydroascorbate reductase were significantly increased, and lipid peroxidation levels were reduced. This suggests that the enzymes involved in the ascorbate-glutathione cycle may play a protective role in the antioxidant process, while CAT, POD, and APX may be associated with the excellent recoverability of *P. pratensis* after drought treatment. Alam et al. (2018) showed that drought tolerance was enhanced by using exogenous melatonin in *F. arundinacea*. Moreover, polyamine plays an important role in regulating stress tolerance in plants. Tang et al. (2020) found that γ-Aminobutyric acid induces NO production and could align with the enhancement of antioxidant defense mechanism. Further, Tan et al. (2022) found that spermine enhanced proline metabolism rather than proline accumulation which could be the main regulatory mechanism for drought tolerance in *A. stolonifera*.

Transcriptome analysis (RNA-Seq) is an accurate and effective tool for evaluating gene expression profiles, providing an excellent view for studying the expression and molecular mechanisms of complex characteristics in plants (Deng et al., 2019). In general, gene expression under stress is a complex network involving multiple genes and therefore, poses a major challenge for pinpointing environmentally responsive genes. RNA-seq has many advantages over microarray techniques, such as the ability to identify new genes or new variable spliceosomes without relying on known gene models, identification of RNA editors, and greater detection sensitivity (Rai et al., 2018). Therefore, RNA-seq is often used for the identification of differentially expressed genes (Figure 3). When comparing the differences in photosynthetic response, Xu et al. (2013) found that the drought-tolerant cultivar of *P. pratensis* had higher levels of ribulose-1,5-bisphosphate carboxylase (Rubisco) and glyceraldehyde phosphate dehydrogenase (GADPH) transcripts, as well as a high photosynthetic rate. The enzymatic activities and gene transcription levels of ribulose phosphate kinase were not significantly different from those of Rubisco. This result suggests that Rubisco-regulated carboxylation and GAPDH-regulated carbon reduction may be the key metabolic processes responsible for genetic variation under drought stress. Rubisco, GAPDH, and Rubisco-activated enzymes are all involved in the recovery of photosynthetic rate to drought stress in *P. pratensis*. Moreover, Xu et al. (2017b) overexpressed the *AtDREB1A* gene in *P. pratensis* and validated its role under drought stress. They found that increased drought resistance of transgenic grass may be associated with an increased accumulation of organic solutes (WSC, proline, and protein) and altered hormone metabolism. Thereafter, Luo et al. (2020) found that a sort of salt-responsive gene in the healing tissue was regulated by microRNAs in *P. pratensis*.

Xie et al. (2015) used high-throughput sequencing to construct a transcriptome database of *Zoysia japonica* under salt stress. Teng et al. (2016) selected the *ZjGRP* gene which is expressed in the roots, stems, and leaves of *Z. japonica* from a database and cloned it in *Arabidopsis*. The translation products were mainly distributed in the nucleus and cytoplasm, and they found that *ZjGRP* expression was induced by NaCl, ABA, MeJA, and SA treatments. Overexpression of this gene caused salt sensitivity in *Arabidopsis*, probably due to its effect on ion transport, osmosis, and antioxidation.

### Response to Heavy Metals

Melatonin can reduce the damage caused by heavy metal ions, chemicals pollutions, extreme temperature or humidity, and high plant oxidative stress. In addition, melatonin can enhance plant resistance to biotic stress. Considering that N-acetyl-5-hydroxybutylamine is converted to melatonin *via* methyltransferase, Luo et al. (2018) cloned the methyltransferase gene, *ZjOMT* from *Z. japonica* and found that this gene was expressed in leaves and roots and was affected by aluminum (Al) stress. The expression of *ZjOMT* in *Escherichia coli* increased the melatonin content by approximately eight-fold in the recombinant strain compared to the control strain, thereby indicating that heterologous expression of *ZjOMT* could increase resistance to Al stress by increasing melatonin content in *E. coli*. Yuan et al. (2018) performed high-throughput sequencing of *A. stolonifera* under cadmium (Cd) stress and screened four families of Cd stress-related transcripts. Their results provide new ideas for further studies on Cd stress response in turfgrass. Ye et al. (2021) proved that alkali stress has a severely inhibitory effect in *C. dactylon* partially due to combined ionic stress and high pH stress. In conclusion, genetic engineering in turfgrass over the recent years has provided rich genetic materials for breeding industrialization.

### Genomic Information and Association Analysis of Turfgrass

In recent years, plant breeding has shifted from phenotypic selection to precise genotypic selection (Varshney et al., 2014). Traditional phenotypic selection methods are expensive and time-consuming (Hajibarat et al., 2015; Saidi et al., 2017). Therefore, determining the molecular mechanisms underlying plant resistance and expression patterns of resistance-associated genes *via* multi-omics studies has become increasingly necessary. The adaptive capacity of plants drives them to escape stresses through physiological and morphological changes. Thus, measuring phenotypic and genotypic changes under different stress treatments can elucidate the molecular mechanisms by which plants respond to stress. The development of sequencing technologies has reduced the cost of high-throughput sequencing, and the widespread demand for scientific research has contributed to the development of NGS which has led to the deposition of millions of sequences in the databank (Nadeem et al., 2018). More and more plants are undergoing whole-genome sequencing, thereby rapidly advancing the process of molecular marker-assisted plant breeding.

Germplasms of turfgrasses are diverse; however, whole genome sequencing was conducted for only a few grasses species owing to their complex genetic background. By December 2021, the whole genomic sequences of *Z. japonica*, *C. transvaalensis, L. perenne* and the model species *Brachypodium distachyon* had been released (International Brachypodium Initiative, 2010; Tanaka et al., 2016; Cui et al., 2021; Frei et al., 2021).

The genus *Zoysia* has 11 species which are mostly tetraploid (2*n* = 40); in general, they are typical warm-season turfgrasses with various morphological and physiological characteristics. Tanaka et al. (2016) used HiSeq and MiSeq platforms to sequence the genome of *Z. japonica* and assembled the whole genomic sequence and predicted 59,271 protein-coding genes. Furthermore, the genome sketches of *Z. matrella* “Wakaba” and *Z. tenuifolia* “Zanpa” were sequenced for comparative genomic analysis (Tanaka et al., 2016). To study the genetic diversity of *Z. japonica*, genome sketches of *Z. japonica* “Kyoto,” *Z. japonica* “Miyagi,” and *Z. matrella* “Chiba Fair Green” were drafted and compared with “Nagirizaki,” “Wakaba,” and “Zanpa” varieties. The results indicated that the polymorphisms in the three species were mainly concentrated between *Z. japonica* and *Z. tenuifolia*, and that these loci tended to be heterozygous. Moreover, the result of heterozygosity of the ANAC102, STO/BBX24, and ANS1 genes, as well as population analysis of genome-wide SSR markers, suggest that *Z. matrella* is most likely an interspecific hybrid between *Z. japonica* and *Z. tenuifolia*.

Cui et al. (2021) sequenced and assembled the genome of a diploid *C. transvaalensis* by using Illumina, Nanopore, BioNano, and Hi-C technologies; genome assembly resulted in 282 scaffolds (~423.42 Mb, N50 = 5.37 Mb), covering 93.2% of the predicted genome size (~454.4 Mb). Considering that selective expansion of gene families often facilitates plant environmental adaptation, Cui et al. (2021) examined 193 heat-shock genes of the HSP70 family from seven species (*C. transvaalensis*, *A. thaliana*, *B. distachyon*, *O. sativa*, *S. bicolor*, *S. viridis*, and *Z. japonica*). Their results revealed higher homology between *C. transvaalensis* and *Z. japonica* compared to other species. *C. transvaalensis* genome encodes more species-specific duplicated genes, providing genetic evidence that *C. transvaalensis* has higher heat tolerance.

Frei et al. (2021) assembled the reference genome of *L. perenne* by an optimal sequencing protocol (Oxford Nanopore Technologies) and obtained a highly complete (2.3 of 2.7 Gb), correct (QV 45), and contiguous (contig N50 and N90 11.74 and 3.34 Mb, respectively) genome assembly. They provided the first high-quality haploid reference assembly for perennial ryegrass and revealed the transposable elements’ dominance and repeated sequences (81.6% of the assembly), and identified 38,868 protein coding genes. Almost 90% of the bases could be anchored to seven pseudomolecules.

*Brachypodium distachyon* is a wild Mediterranean and Middle Eastern herb with a small genome size, simple growth conditions, and a short life cycle. Its genome size is approximately 272 Mb, and it is closely related to economically important crops, such as wheat, barley, maize, and sorghum. As *B. distachyon* was first to be sequenced entirely, it is treated as a model plant for functional genomic studies in the Subfamily Pooideae, which contains a large number of economically important crops.

## Suggestions on Resistance Breeding in Turfgrass

Many countries give equal importance to planting and animal husbandry in the overall development of agriculture. Developed countries are constantly developing in the direction of science and modernization in the management and construction of grassland. Although the rapid development in the grass industry in China has filled the gap in turfgrass research, it is still behind that of the developed countries. In the future, we should focus more on the development of talent in turf, professionals in turf cultivation and management, application of breeding and biotechnology, as well as pest and disease control, comprehensively and healthily.

However, with the increasing demand for quality and yield in urban turfs, there is an urgent need to produce new varieties with distinctive colors or phenotypes that are adapted to environmental pressures. Therefore, hastened breeding process and genetic research, either *via* traditional techniques or molecular breeding, are necessary to meet these demands. However, there is a misalignment between scientific research and practice, as field workers find suitable mutants but do not know how to utilize them, while laboratory researchers are constrained by the lack of suitable mutants for their research. There is therefore a great need to build a resistance breeding platform of turfgrass to break the barriers between turfgrass research and industry. With advances in sequencing technology and reduced costs, vast amounts of data have been accumulated on genetic variations, in genome, transcriptome, and metabolome. Huge databases will lay the foundation for transgenic breeding and genome editing in turfgrass. Although, many genes associated with target traits can be selected from bioinformatic analyses, it is difficult to speed up the process of identification. It is, therefore, necessary to combine molecular data with breeding and phenotypic data for analysis. Breaking the barriers between industry, academia, and research will help in the advancement of turfgrass research and industry.

### Improving Turfgrass Resistance Through Distant Hybridization

Distant hybridization also known as cross-breeding occurs between different species, genera or more distantly related plant species and is a key means of overcoming reproductive isolation. Distant hybridization can combine biological characteristics of distantly related species, break species restrictions, amplify genetic variation, lead to phenotypic and genotypic variations, and create new variants or species (Liu, 2014). Using distant hybridization, genetic resources for commercial characteristics (e.g., disease resistance, insect resistance, high stress resistance, high quality, etc.) from other species can be introduced into the target species. However, some of the limitations of distant hybridization are incompatibility of crosses, hybrid decay or sterility, segregation of hybrid progeny, and time-consuming acquisition of stable genetic material. Regardless, the introduction of exogenous genes through distant hybridization has become an important means of expanding genetic variation among plants (Sharma and Gill, 1983). The cross-border expression of resistance genes often leads to unexpected results. In the 1990s, researchers transferred the antifreeze protein gene (AFP) from *Pseudopleuronectes arnericanus* into tomatoes and found that the transgenic tomatoes not only stably transcribed the AFP mRNA but also expressed AFP, and that tissue extracts from this transgenic tomato were effective in preventing the growth of ice crystals under freezing conditions (Hightower et al., 1991). More notably, the genomes of extreme habitat organisms are more likely to be screened for novel genes or new functional and structural domains imparting environmental adaptation mechanisms. The presence of a species-specific META domain in the salt resistance gene Hal2 of the yeast *Aureobasidium pullulans* improved the salt resistance of the transgenic *Arabidopsis* and greatly increased its drought resistance (Gostinčar et al., 2011). Therefore, the selection of specific functional genes for application in plants is a novel strategy to improve turfgrass stress tolerance.

Isotope marker relative quantification (iTRAQ) and transcriptome techniques can be used to analyze the mechanisms of distant hybrid incompatibility barriers and embryo rescue, and *in vitro* fertilization can be used to overcome pre-fertilization reproductive barriers (He et al., 2020). Polyploidy induction can be used as an intermediate means of breeding, combined with cell fusion techniques, gene editing techniques, chromosome fragment substitution, and other techniques that can be used to overcome hybridization barriers. Cross-breeding with different inbred species, using superior characteristics from different populations, as well as using mutagenesis to create mutants, can create more plants with variations in chromosome structure, such as the cloning of the *Yr28* and *Pm21* genes in wheat (Fu et al., 2009; Zhang et al., 2019). Gene covariance can be verified using the chromosomal interval where the super genes of the model plant have been cloned and the above-mentioned small fragment interval, and then homologous cloning of the genes from closely related plants.

### Genomic Data Mining and Exploitation

Most turfgrass species are heterozygous and have a complex genetic background. Therefore, SSR markers are not effective in amplifying allelic fragments in samples at high ploidy levels. The presence of a band or a fragment indicates the presence of an allele on the chromosome forming the homologous cluster, whereas the absence of a fragment indicates that the same allele is not present on any chromosome. SSR markers can be used for GWAS analysis, but it is limited primarily due to the lack of SSRs in microsatellite databases (Gebhardt et al., 2004; Guo et al., 2012). In GWAS analysis, the use of SNP markers is more advantageous as the expected heterozygosity (He) of SNP markers is lower than that of SSR markers and yields better results in population structure evaluation analysis for most crops. In addition, SNPs are distributed throughout the genome and are cheaper than SSRs. Some species, such as chrysanthemum, carnation, pinto bean, chickpea, and potato have already been applied for marker-assisted breeding (Hajibarat et al., 2015; Saidi et al., 2018). One of the most important reasons for using GWAS is that it has high-resolution mapping, a large volume of data, and better identification of rare alleles (Guo et al., 2012). However, this technique is limited by the genomic information availability. Although most species of turfgrass have more complex genetic backgrounds and long durations of population generation, there are only a few reports on GWAS analysis in turfgrass.

Meanwhile, SCoT and CDDP markers have received more attention in recent years, as they do not require genome sequence databases (Andersen and Lubberstedt, 2003), while molecular markers developed based on conserved regions can also be identified across species (Al-qurainy et al., 2015), and markers developed based on functional genes have a stronger advantage over random markers for trait association (Andersen and Lubberstedt, 2003).

### Building a Turfgrass Resilience Breeding Platform

Although there is relatively little genomic data on turfgrass, the increasing accumulation of transcriptomic data will facilitate research on important traits, such as salt tolerance, cold tolerance, and disease resistance. Identification of gene function is a key prerequisite, while a stable genetic transformation system is the foundation. Currently, the Turf Research Institute of Beijing Forestry University has constructed stable genetic transformation systems based on gene gun transformation methods (Qi et al., 2005; Xin et al., 2006) and *Agrobacterium*-mediated methods (Dai, 2013) for major turfgrass species, such as *P. pratensis* and *Z. japonica*, focusing on improving stress resistance in turfgrass. Although research on gene editing and CRISPR/Cas9 is gradually being strengthened, there are some limitations on policy and experimental conditions for field validation and utilization of transgenic strains.

Research on key characteristics, whether by traditional or molecular breeding, is used to obtain superior progeny with the desirable target trait. Each method has its advantages and disadvantages and is unlikely to accelerate the breeding process if applied independently. Therefore, a resistance breeding platform should be constructed to integrate a range of approaches and research ideas, including breeding techniques, herbicide resistance mechanisms, molecular mechanisms underlying stress tolerance and disease resistance, and the development of molecular markers for identifying important characteristics, to facilitate the breeding of new resistant turfgrass varieties.

## Conclusion

In recent years, the amount of grass species used for turfgrass establishment has increased rapidly and led to an increase in the area under urban landscaping in China. Hundreds of species have been introduced in China and new varieties are available every year. Further, the introduction of good quality turfgrasses has contributed to the development of China’s turf industry. The continuous improvement of the molecular breeding system and scientific research has largely stimulated domestic researchers to continue to breed turfgrass species suitable for China’s climatic conditions. At present, there is a huge market for grass seeds in China, and with a green space coverage rate of <10% and an urban green space of <1.6 square meters per person, more investment is needed to change the status. In addition, the landscape of large factories, construction of sports grounds, soil and water conservation projects for highways, railways, and river banks, and desertification treatments require large amounts of high-quality grass seeds. These potential markets provide a good opportunity for the development of resistance breeding for turfgrass in China.

## Author Contributions

LJ wrote the manuscript. HL suggested the concept of the manuscript and worked in the manuscript structure and language and contributed to the overall look of the manuscript. All authors contributed to the article and approved the submitted version.

## Funding

This research was funded by National Natural Science Foundation of China, grant number 31971770.

## Conflict of Interest

The authors declare that the research was conducted in the absence of any commercial or financial relationships that could be construed as a potential conflict of interest.

## Publisher’s Note

All claims expressed in this article are solely those of the authors and do not necessarily represent those of their affiliated organizations, or those of the publisher, the editors and the reviewers. Any product that may be evaluated in this article, or claim that may be made by its manufacturer, is not guaranteed or endorsed by the publisher.
